# Magneto-Mechanical Coupling in Magneto-Active Elastomers

**DOI:** 10.3390/ma14020434

**Published:** 2021-01-17

**Authors:** Philipp Metsch, Dirk Romeis, Karl A. Kalina, Alexander Raßloff, Marina Saphiannikova, Markus Kästner

**Affiliations:** 1 Institute of Solid Mechanics, Technische Universität Dresden, 01062 Dresden, Germany; philipp.metsch@tu-dresden.de (P.M.); karl.kalina@tu-dresden.de (K.A.K.); alexander.rassloff@tu-dresden.de (A.R.); 2 Leibniz-Institut für Polymerforschung Dresden e.V., Hohe Strasse 6, 01069 Dresden, Germany; grenzer@ipfdd.de; 3 Dresden Center for Computational Materials Science (DCMS), Technische Universität Dresden, 01062 Dresden, Germany

**Keywords:** magneto-active elastomers, magneto-mechanical coupling, magneto-striction, magneto-deformation

## Abstract

In the present work, the magneto-mechanical coupling in magneto-active elastomers is investigated from two different modeling perspectives: a micro-continuum and a particle–interaction approach. Since both strategies differ significantly in their basic assumptions and the resolution of the problem under investigation, they are introduced in a concise manner and their capabilities are illustrated by means of representative examples. To motivate the application of these strategies within a hybrid multiscale framework for magneto-active elastomers, their interchangeability is then examined in a systematic comparison of the model predictions with regard to the magneto-deformation of chain-like helical structures in an elastomer surrounding. The presented results show a remarkable agreement of both modeling approaches and help to provide an improved understanding of the interactions in magneto-active elastomers with chain-like microstructures.

## 1. Introduction

Field-controllable functional polymers represent a relatively new class of applied materials exhibiting a strong coupling of mechanical and additional—e.g., electric, magnetic, or thermal—external fields. The application of these external fields influences the interactions between different local material phases and causes an evolution of the microstructure. A prominent example of field-controllable functional polymers are magneto-active elastomers (MAEs) which feature mechanical moduli that can be enhanced under an applied magnetic field [1,2,3,4,5,6,7,8,9,10] as well as the ability for magnetically induced deformations [2,3,11] and actuation stresses. These properties make MAEs attractive for a variety of technical implementations: up to now, applications for actuators and sensors [12,13,14], energy harvesting [15,16,17], micro-robots [18], and -pumps [19] as well as prosthetic and orthotic devices with tunable stiffness [20] have been proposed.

In all of these applications, the effective magneto-mechanical material behavior is of special interest. To this end, an in-depth understanding of the materials’ structure-property relationships is required. MAEs typically represent composite materials, in which micron-sized magnetizable particles are embedded into a compliant polymer network [21,22,23,24,25,26]. The flexibility of polymer sub-chains between cross-links allows for a considerable degree of particle rearrangement under strong magnetic fields. This is especially true for sufficiently soft matrices, in which the particles are prone to organize themselves into elongated microstructures, that significantly influence the coupled magneto-mechanical properties of MAEs. The simultaneous consideration of inhomogeneous magnetic and mechanical fields and their interaction with the underlying, evolving microstructure is the particular challenge in the modeling of MAEs.

**Figure 1:**
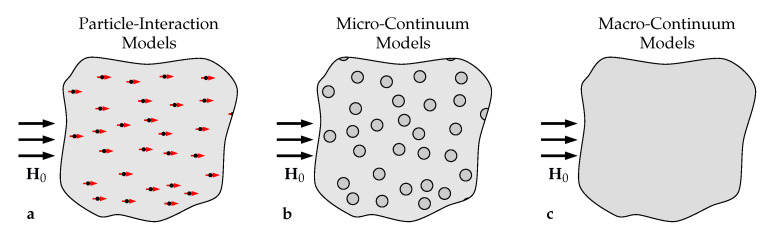
Illustration of different modeling strategies for MAEs: (**a**) particle–interaction models with dipolar magnetic particles (red arrows) distributed in an elastomer matrix (light gray background), (**b**) micro-continuum models with fully resolved particles (dark gray circles) within the same matrix, and, (**c**) homogeneous macro-continuum models with no resolution of the underlying microstructure.

In order to provide a better understanding of the effective material behavior of MAEs, different theoretical approaches have been presented in the literature over the last few years. Following the schematic illustrations in Figure 1, they can be divided into particle–interaction as well as micro- and macro-continuum models. With few exceptions [27,28], particle–interaction models, see Figure 1a, are based on a calculation of the effective MAE behavior from overall energetic expressions that can be found using the dipole approximation, i.e., by not resolving the local magnetic fields [29,30,31,32]. Consequently, the approach allows for considering MAE specimens comprising a large number of particles with little computational effort. On the other hand, the method provides only an approximate description of the particle interactions within the material and, thus, is especially suited to characterize dilute systems with a low particle–volume fraction. As indicated in Figure 1b, micro-continuum models pursue a different approach: here, local magnetic and mechanical fields are resolved explicitly using a continuum formulation of the magneto-mechanical boundary value problem [33,34,35]. Since this yields a system of fully coupled, nonlinear partial differential equations which require computationally demanding numerical methods—such as a finite element (FE) analysis—for their solution, only comparably small MAE samples as well as representative microstructures in the surrounding of a material point can be considered. To this end, the approach allows for identifying mechanisms leading to magnetically induced deformations and enhanced mechanical moduli on the microstructural level but requires an appropriate computational homogenization procedure to predict the effective material behavior [36,37,38,39]. In macro-continuum approaches, the MAE is modeled as a homogeneous continuum in which microstructural information are captured via suitable coupling terms; see Figure 1c. Here, a phenomenological material model is typically fitted to data obtained from experiments [40,41] or more resolved, microscopic analyses [42,43,44]. Due to its phenomenological nature, this strategy cannot provide any understanding of microscopic mechanisms which drive the materials’ effective response, but—as it allows a consideration of realistic MAE samples under complex loading conditions—yields relevant information on macroscopic shape effects [45].

Considering experimental investigations on MAEs, their effective properties seem to be ambiguous. Especially for the magnetically induced deformation—a phenomenon which here is referred to as magneto-deformation to avoid confusion with the intrinsic magneto-striction of single-phase materials—samples with comparable shapes and particle–volume fractions are reported to show either a contraction [11,46] or an elongation [40,47,48] in the direction of the applied magnetic field. This shows that micro- and macrostructural effects require to be taken into account for an understanding of the material. Consequently, both structural levels have to be captured in the pursued modeling strategy. To allow this, a combination of all of the aforementioned approaches is advantageous: the microscale can be considered performing fast analyses with particle–interaction models while using the obtained results for a fitting of a macro-continuum model enables investigations of realistic MAE samples under complex loading conditions. However, due to their accuracy, micro-continuum models are still required to define the limits of the simplified particle–interaction approach—only by comparing both strategies for the range of targeted applications and microstructures, a reasonable characterization of MAEs can be ensured.

On the way towards the development of such a hybrid multi-scale approach, the main focus of the current contribution is a comprehensive comparison of the two micro-modeling strategies. Up to now, these approaches mainly coexisted and have only been compared for simplified two-dimensional MAEs in a preceding article of the authors [49]. Here, this work is extended to the general three-dimensional case with an emphasis on chain-like microstructures. An analysis of ideal helical structures allows for investigating the magnetic response of chains with different angles and distances between the individual particles and, thus, facilitates an identification of distributions which are more prone to positive magneto-deformation, i.e., an elongation, than others. In view of the fact that structured MAEs are of significant interest within the research community, these results can help to provide an improved understanding of realistic chain-like structures.

The paper is organized as follows: in Section 2, the applied micro-modeling strategies, i.e., the micro-continuum and the particle–interaction models, are presented—relevant equations are specified and the capabilities of the individual approaches are summarized using representative examples. In the subsequent Section 3, the comparison of both approaches regarding their predictions for the magnetically induced deformation of helical chains is performed: after the problem under consideration is outlined, the results and their implications for the modeling of MAEs are discussed. Finally, the paper is concluded by a short summary in Section 4.

## 2. Micro-Modeling Strategies

**Figure 2:**
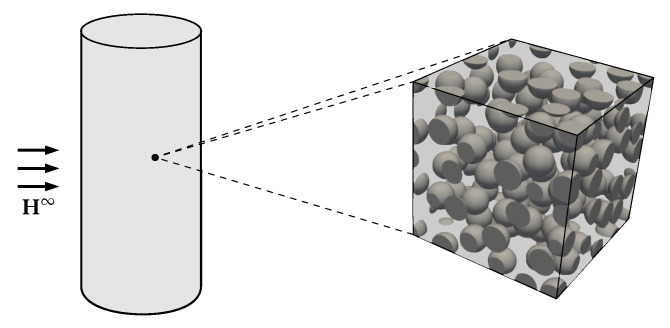
Illustration of an MAE sample: a macroscopic cylindrical sample in an external magnetic field $H^{\infty }$ with an exemplary random microstructure at each material point.

In order to clarify the common basis of the models presented in this section, Figure 2 can be used: it illustrates an MAE sample with a cylindrical shape which is frequently used in experiments [50,51,52]. In the vicinity of each material point, the microstructure is assumed to consist of stiff, magnetizable particles surrounded by a soft and non-magnetizable elastomer matrix. In Figure 2, a monodisperse random microstructure with spherical particles is shown—however, this distribution can vary depending on the used materials and the sample preparation process [11,53].

The experimentally observable magneto-deformation as well as changes of the samples’ moduli are a result of mutual particle interactions caused by an external magnetic field $H^{\infty }$. Since, within this contribution, only magnetically soft materials are of interest, the local magnetization can be described via functions of the form

(1) $$M\left(r\right)=M\left(|H(r)|\right)\frac{H(r)}{\left|H(r)\right|}$$

which relate the magnetization M and magnetic field H at any material point r. Note that M and H are always aligned—their connecting function M represents any saturating function, e.g., of Langevin-, hyperbolic-tangent- or Frölich–Kennelly-type, which only depends on the norm of the magnetic field. For the mechanical properties of the matrix material, a purely elastic behavior is assumed. Its implementation within the individual approaches is presented in the subsequent subsections.

### 2.1. Micro-Continuum Model

The foundation of the micro-continuum model presented here is the work of de Groot and Suttorp [54]. Therein, long- and short-range contributions of atomic interactions are investigated for various macroscopic physical quantities—the resulting field equations feature additional coupling terms and are obtained by performing statistical averages to a level where the continuum hypothesis can be considered valid. For the interaction of stationary magnetic and mechanical fields, the governing equations will be briefly introduced, in the following.

#### 2.1.1. Governing Equations

The magnetic part of the coupled problem requires Maxwells equations to be solved [55]. Assuming a material body which is free of any current densities and surface currents, they are given by

(2) $$\begin{array}{cc} \nabla \;\cdot \;B & =0 \end{array}$$

(3) $$\begin{array}{cc} \nabla \times H & =0 \end{array}$$

and their associated jump conditions [56]. Here, B and H represent the magnetic induction as well as the magnetic field of a material point in the current configuration [57]—they are linked by the magnetization M via the equation

(4) $$B=\mu_{0}\left(H+M\right)$$

with $\mu_{0}$ being the permeability of free space. As shown in [56,57], the coupling of the mechanical fields into the magnetic equations can be pointed out by defining the reference fields using the deformation gradient F and its determinant *J*:

(5) $$H_{0}=F^{T}\;\cdot \;H\;,\;B_{0}=JF^{-1}\;\cdot \;B\;\mathrm{and}\;M_{0}=F^{T}\;\cdot \;M.$$

Finally, a scalar potential approach with H=−∇φ is applied within this contribution to reduce the number of unknowns and equations to be solved [55].

For the mechanical part of the problem, the existence of magnetic field yields additional body force $f^{\mathrm{pon}}={\left(\nabla B\right)}^{T}\;\cdot \;M$, couple $c^{\mathrm{pon}}=M\times B$ and power $p^{\mathrm{pon}}=M\;\cdot \;\dot{B}$ densities which have to be accounted for within the individual balance equations. Especially the additional couple density has severe consequences for the solution of the coupled magneto-mechanical boundary value problem: the mechanical stress σ is not necessarily symmetric. In order to ease the computation, the magnetic body force density can be expressed as the divergence of a ponderomotive stress via $f^{\mathrm{pon}}=\nabla \;\cdot \;\sigma^{\mathrm{pon}}$ with

(6) $$\sigma^{\mathrm{pon}}=\frac{1}{\mu_{0}}\left[BB-\frac{1}{2}\left(B\;\cdot \;B\right)I\right]+\left(B\;\cdot \;M\right)I-BM$$

and I being the identity tensor [57]. This mathematical trick facilitates the introduction of the symmetric total stress $\sigma^{\mathrm{tot}}=\sigma +\sigma^{\mathrm{pon}}$ and—if vanishing mechanical body force densities are assumed—leads to the following balance of linear momentum:

(7) $$\nabla \;\cdot \;\sigma^{\mathrm{tot}}=0.$$

Since thermal effects are not within the scope of this work, the balance of the internal energy yields no additional information and is omitted here for brevity, see [58] for further information. For the derivation of thermodynamically consistent constitutive models, only the Clausius–Duhem inequality—as a consequence of the entropy balance and the second law of thermodynamics—is still required. With $C=F^{T}\;\cdot \;F$ being the right Cauchy–Green deformation tensor, the amended free energy

(8) $$\Omega =\Psi -\frac{\mu_{0}}{2}JC^{-1}\;:\left(H_{0}H_{0}\right)$$

extends the Helmholtz free energy Ψ by free field magnetic contributions [57]. Using this relation, the Clausius–Duhem inequality can be stated as:

(9) $$-\dot{\Omega }+J\sigma^{\mathrm{tot}}:\left(\dot{F}\;\cdot \;F^{-1}\right)-B_{0}\;\cdot \;{\dot{H}}_{0}\geq 0.$$

#### 2.1.2. Constitutive Models

In order to ensure constitutive models that are not only thermodynamically consistent but also objective, quantities of the reference configuration are chosen as independent variables. Here, the right Cauchy–Green deformation tensor C as well as the reference magnetic field $H_{0}$ are used. By performing an evaluation of the Clausius–Duhem inequality (9) according to the procedure of Coleman and Noll [59], the constitutive relations for the magnetic induction and the total stress can be found:

(10) $$B=-J^{-1}F\;\cdot \;\frac{\partial \Omega }{\partial H_{0}},\;\sigma^{\mathrm{tot}}=2J^{-1}F\;\cdot \;\frac{\partial \Omega }{\partial C}\;\cdot \;F^{T}.$$

Since both constituents in MAEs, the stiff magnetizable particles, and the non-magnetizable elastomer matrix show intrinsic magneto-mechanical coupling effects that are vanishingly small if compared to the compounds coupling behavior, the free energy term in Equation (8) can be split into purely magnetic and mechanical contributions: $\Psi \left(H_{0},C\right)=\Psi^{\mathrm{mag}}\left(H_{0}\right)+\Psi^{\mathrm{mech}}\left(C\right)$.

For the latter, an isotropic hyperelastic energy function is adequate—it allows for accounting for potentially large deformations of the matrix material and to capture the resulting rotations of the particles. The choices for such functions vary from simple neo-Hookean to elaborate Ogden models which allow for accurately describing a nonlinear elastic behavior over a wide range of deformations [60]. For the different results presented in this contribution, the following energetic contributions are applied:

(11a) $$\begin{array}{cccc} & \mathrm{Ogden}:\; & \Psi_{1}^{\mathrm{mech}}\left(C\right) & =\sum_{p=1}^{N}\frac{\mu_{p}}{\alpha_{p}}\left(\sum_{\beta =1}^{N_{\lambda }}{\nu_{\beta }\lambda_{\beta }^{\mathrm{iso}}}^{\alpha_{p}}-3\right)+\frac{\kappa }{4}\left[I_{3}-\ln\left(I_{3}\right)-1\right] \end{array}$$

(11b) $$\begin{array}{cccc} & \mathrm{Mooney}-\mathrm{Rivlin}:\; & \Psi_{2}^{\mathrm{mech}}\left(C\right) & =\frac{1}{2}\left[\mu_{1}\left(I_{1}^{\mathrm{iso}}-3\right)-\mu_{2}\left(I_{2}^{\mathrm{iso}}-3\right)\right]+\frac{\kappa }{4}\left[I_{3}-\ln\left(I_{3}\right)-1\right] \end{array}$$

(11c) $$\begin{array}{cccc} & \mathrm{neo}-\mathrm{Hooke}:\; & \Psi_{3}^{\mathrm{mech}}\left(C\right) & =\frac{\mu }{2}\left[I_{1}-\ln\left(I_{3}\right)-3\right]+\frac{\lambda }{4}\left[I_{3}-\ln\left(I_{3}\right)-1\right]. \end{array}$$

Therein, the quantities $I_{k}$ and $I_{k}^{\mathrm{iso}}$ represent the principle invariants of C and its isochoric counterpart $C^{\mathrm{iso}}=J^{-\frac{2}{3}}C$, $\lambda_{\beta }^{\mathrm{iso}}$ are the eigenvalues of $F^{\mathrm{iso}}=J^{-\frac{1}{3}}F$ with algebraic multiplicity $\nu_{\beta }$ and the sets $\left\{\mu_{p},\;\alpha_{p},\;\kappa \right\}$, $\left\{\mu_{1},\;\mu_{2},\;\kappa \right\}$ as well as $\left\{\mu ,\;\lambda \right\}$ are corresponding material parameters which are specified within the individual examples.

For the magnetic contribution of the Helmholtz free energy, $\Psi^{\mathrm{mag}}=0$ holds within the matrix material, while the experimentally observed magnetization curves of carbonyl iron powder and nickel particles as described in [61,62] are applied to capture the nonlinear magnetization behavior of the particles. Thus, $\Psi^{\mathrm{mag}}$ is given by

(12a) $$\begin{array}{cccc} & \mathrm{Langevin}:\; & \Psi_{1}^{\mathrm{mag}}\left(H_{0}\right) & =-\frac{\mu_{0}M_{s}}{\zeta }\left[\ln\left(\sinh(\zeta H_{0})\right)-\frac{1}{\zeta H_{0}}\right]\;\mathrm{and} \end{array}$$

(12b) $$\begin{array}{cccc} & \mathrm{Hyperbolic}\;\tan\mathrm{gent}:\; & \Psi_{2}^{\mathrm{mag}}\left(H_{0}\right) & =-\frac{\mu_{0}M_{s}}{\xi }\ln\left(\cosh\left(\xi H_{0}\right)\right) \end{array}$$

with $\left\{M_{s},\;\zeta \right\}$ and $\left\{M_{s},\;\xi \right\}$ being sets of associated saturation magnetizations and scaling parameters that are used in the examples. Keeping in mind that the particles are almost rigid, if compared to the elastomer matrix, a specification of the magnetization function introduced in Equation (1) yields relations which solely depend on the norm $H_{0}$ of the magnetic field $H_{0}$:

(13a) $$\begin{array}{cccc} & \mathrm{Langevin}:\; & M_{1}\left(H_{0}\right) & =M_{s}\left[\coth\left(\zeta H_{0}\right)-\frac{1}{\zeta H_{0}}\right], \end{array}$$

(13b) $$\begin{array}{cccc} & \mathrm{Hyperbolic}\;\mathrm{tangent}:\; & M_{2}\left(H_{0}\right) & =M_{s}\tanh\left(\xi H_{0}\right). \end{array}$$

#### 2.1.3. Computational Homogenization Framework

In order to be able to determine physically meaningful macroscopic quantities from numerical simulations on the microscopic scale, an adequate scale transition has to be performed. Following the work of Hill [63] and its extension to the coupled magneto-mechanical boundary value problem [36], the Hill–Mandel condition, i.e., the equivalence of the macroscopic and averaged microscopic energies, is applied here. If the averaging process of a microscopic quantity $\left(\;\cdot \;\right)$ is indicated by $\langle \left(\;\cdot \;\right)\rangle$ and its macroscopic counterpart is denoted as $\bar{\left(\;\cdot \;\right)}$, it is given by

(14) $$\langle P^{\mathrm{tot}}:\dot{F}\rangle -\langle B_{0}\;\cdot \;{\dot{H}}_{0}\rangle ={\bar{P}}^{\mathrm{tot}}:\dot{\bar{F}}-{\bar{B}}_{0}\;\cdot \;{\dot{\bar{H}}}_{0}.$$

Therein, $P^{\mathrm{tot}}=JF^{-1}\;\cdot \;\sigma^{\mathrm{tot}}$ is the first Piola–Kirchhof stress tensor. Within the FE simulations, the fulfillment of the condition is ensured by making use of representative volume elements (RVEs) for which periodic boundary conditions are applied [56].

#### 2.1.4. Model Applications

In the following, the versatility of the presented micro-continuum approach will be outlined by means of three representative examples. In order to ensure that the strategy is eligible for the modeling of the strongly coupled magneto-mechanical behavior of MAEs, it is validated with experimental data for a simplified MAE specimen in the beginning. Afterwards, recent findings for the field-dependent behavior of MAEs with different ideal and random microstructures are presented. Since the micro- and macroscales need to be considered to capture all effects of realistic MAE samples, a first approach towards the development of a hybrid multiscale modeling strategy by fitting a macro-continuum model with data generated from micro-continuum simulations is briefly summarized as a last example.

##### Model Validation by Means of a Simplified MAE Specimen

While the material behavior of the MAE’s constituents is often well-known, macroscopic effects of inhomogeneous magnetic and mechanical fields make an experimental characterization of their compound behavior almost impossible [42,64]. To this end, a proper validation of the micro-continuum model can only be performed with well-defined, simplified MAE samples under controlled loading scenarios. In a previous study, this procedure was applied for systems with only few magnetizable particles [62]. It allows for a detailed analysis of the sample behavior with a limited number of influencing factors. Here, the results for the three-particle sample in the aforementioned work are summarized.

**Figure 3:**
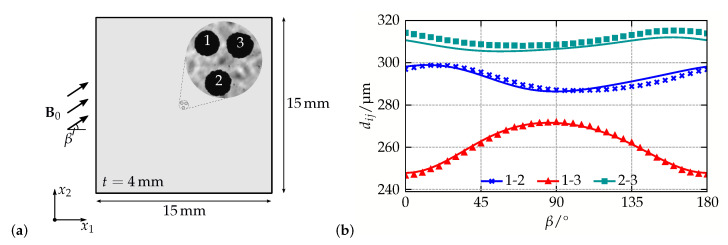
Model validation with a simplified three-particle system: (**a**) schematic illustration of the system with microscopic pictures of the magnetizable particles, and (**b**) comparison of experimental (markers) and numerical (solid lines) results for the course of the inter-particle distances $d_{ij}$ in a magnetic field with varying angle β. The initial distances of the particles are $d_{12}=298\;\mu m$, $d_{13}=269\;\mu m$ and $d_{23}=312\;\mu m$. Data taken from [62].

A schematic illustration of the system under consideration is shown in Figure 3a: three magnetizable nickel particles with diameters of approximately 200 μm were placed into the center of a Polydimethylsiloxane (PDMS) matrix with a quadratic cross section of 15 mm length and a thickness of 4 mm, see [62] for further information on the sample generation. During the experiment, a magnetic field $B_{0}$ with norm $B_{0}=170\;mT$ was applied. While its angle β was changed in steps of $\Delta \beta =5{}^{\circ }$, the inter-particle distances $d_{ij}$ of the magnetizable inclusions were tracked using a microscope. The results are shown in Figure 3b.

Within the FE simulations, the experimental setup was mimicked as closely as possible: only the shape of the magnetizable inclusions has been approximated by spheres—this results in a symmetry of the specimen with respect to the $x_{1}$-$x_{2}$-plane. For the FE simulations, the displacement of all outer specimen boundaries was fixed, while inhomogeneous Dirichlet boundary conditions were used for the magnetic scalar potential to apply the external magnetic field. Regarding the constitutive behavior, the energetic contributions introduced in Equations (11b) and (12b) have been used. For the magnetizable nickel particles, experimental investigations using a novel approach with a superconducting quantum interference device have shown that $M_{s}=314.5\;kA/m$ and $\xi =1.886\cdot 10^{-2}\;m/kA$ are a reasonable choice to describe their magnetization curve, while $\mu_{1}^{p}=76\;G\mathrm{Pa}$, $\mu_{2}^{p}=0$, and $\kappa^{p}=164\;G\mathrm{Pa}$ are applied to account for their elastic behavior. Since no full experimental characterization of the elastomer matrix was available, the numerical simulations have been performed for $\nu^{m}=0.49$ and various matrix moduli—the optimal value $E_{\mathrm{opt}}^{m}=6842\;\mathrm{Pa}$ was determined in a least-squares sense by comparing the experimental and numerical predictions for every distance and angle.

The comparison of the experimental and numerical results for the best fit in Figure 3b shows a very good qualitative and quantitative agreement: for all inter-particle distances $d_{ij}$, the sample behavior is captured by the presented micro-continuum approach which demonstrates that it represents an adequate framework to describe microstructural interactions in MAEs. Variations of the particles initial positions that were also performed in [62], indicate a sensitivity of the simulations with regard to the sample geometry. To this end, the systematic error for $d_{23}$ and the small deviations for the distance of particles 1 and 2 can be eliminated by incorporating data from micro-tomography measurements [65] for the generation of more accurate simulation models.

##### Field-Dependent Behavior of MAE Microstructures

As a field-induced change of the macroscopic stiffness, the magneto-rheological (MR) effect is one of the most frequently investigated effects in MAEs [1,8,66,67]. In such experiments, MAE samples with different shapes and microstructures are typically exposed to a shear deformation for varying external magnetic fields: by comparing the modulus to the one of the magnetically unloaded sample, the MR effect is determined. On the microstructural level, the effect can be ascribed to attractive and repulsive forces between the interacting magnetizable particles which means that—apart from macroscopic shape effects due to, e.g., inhomogeneous magnetic fields—different particle distributions must entail differences in the materials MR effect as well.

In a recent study on the field-dependent behavior of MAEs, two microstructures have been investigated exemplarily regarding their influence on the MR effect—a lattice-like simple cubic and a more realistic random microstructure [68]. For the analysis, the experimental setup was mimicked within a finite element simulation: representative volume elements for the materials’ microstructure were exposed to a macroscopic magnetic field ${\bar{H}}_{0}$ while their macroscopic deformation was fixed. At the end of each load step, a small shear deformation $\bar{\gamma }$ was applied to determine the shear components of the macroscopic, i.e., compound, stiffness tensor. Using the nominal macroscopic mechanical stress tensor $\bar{P}$ and a loading of the following form:

(15) $$\left[{\bar{H}}_{0}\right]=\left[\begin{array}{c} {\bar{H}}_{0}\\ 0\\ 0 \end{array}\right],\;\left[\bar{F}\right]=\left[\begin{array}{ccc} 1 & 0 & 0\\ \bar{\gamma } & 1 & 0\\ 0 & 0 & 1 \end{array}\right],$$

the analyzed macroscopic shear component $\bar{G}$ and its relative change $\Delta \bar{G}$ are defined by:

(16) $$\bar{G}\;\left({\bar{H}}_{0}\right)=\frac{{\bar{P}}_{21}\left({\bar{H}}_{0},\bar{\gamma }\right)-{\bar{P}}_{21}\left({\bar{H}}_{0},0\right)}{\bar{\gamma }}\;\mathrm{and}\;\Delta \bar{G}\;\left({\bar{H}}_{0}\right)=\frac{\bar{G}\left({\bar{H}}_{0}\right)-\bar{G}\left(0\right)}{\bar{G}\left(0\right)}.$$

**Figure 4:**
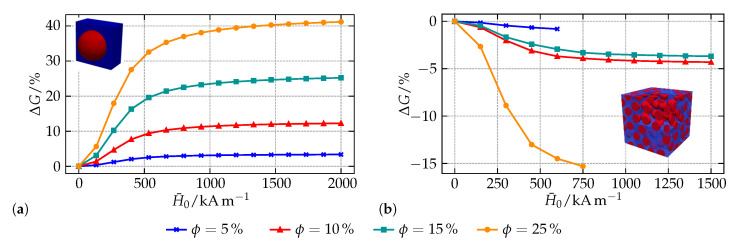
Simulation results for the MR effect in MAEs with different microstructures: (**a**) lattice-like cubic microstructure, and (**b**) random microstructure with varying particle-volume fractions. Data taken from [68].

To obtain the results depicted in Figure 4, the energy functions (11c) and (12a) have been employed. The stiff magnetizable particles are characterized by $M_{s}=868\;kA/m$, $\zeta =2.18\cdot 10^{-2}\;m/kA$ as well as $\lambda^{p}=121\;G\mathrm{Pa}$ and $\mu^{p}=81\;G\mathrm{Pa}$. For the elastomer matrix, $\lambda^{m}=1644\;k\mathrm{Pa}$ and $\mu^{m}=34\;k\mathrm{Pa}$ are applied. As can be seen from the results for the cubic microstructure in Figure 4a, a field-induced stiffening is found for all particle-volume fractions ϕ. All curves show the typical behavior of an initially quadratic course with a saturation for higher magnetic fields [56,69]. Qualitatively, the results are in good agreement with findings of other authors [70,71]. Quantitatively, an increase of the modulus by up to 40% is observed with moderate particle-volume fractions—this effect can even be increased by considering a softer elastomer matrix as it is often used in realistic MAE samples.

Regarding the RVEs comprising 100 randomly placed inclusions with the same particle-volume fractions in Figure 4b, the situation is different. Although many experimental as well as numerical investigations report a field-induced increase of the materials modulus for such microstructures, a decreasing stiffness is found within this study. From the qualitative point of view, the course of the curves is similar to the one observed for the cubic microstructure. However, it is apparent that the MR effect is not systematic for the analyzed RVEs: the microstructure with ϕ = 10% shows a bigger effect than the one with ϕ = 15%. As will be pointed out in the subsequent example, recent findings of the authors show that—on the microstructural level—the effects in MAEs are driven by clusters of interacting particles which are very close to each other. Minimum distances, here 27.5% of the particle diameter, which act as a limit to impede intersecting particles during the random placement algorithm in the geometry generation can, as in this and other examples [72], entail such an unsystematic behavior, i.e., produce microstructures that are random but apparently not representative. Concerning the missing data points for ϕ = 5% as well as ϕ = 25% another drawback of the applied modeling strategy in this example can be seen: for increasing magnetic fields, the simple neo-Hookean material model (11c) is not able to resist the attractive forces between close inclusions, see [73] for further information. To this end, the numerical simulations start to fail.

##### Macroscopic Model Calibration Using a Decoupled Multiscale Framework

To facilitate a full characterization of MAEs on the microscopic and macroscopic scales, a multiscale framework is required. In the following, the procedure according to [42,68], where a macro-continuum model is calibrated from micro-continuum simulations for the simplified two-dimensional case, is briefly presented. The framework represents a decoupled multiscale scheme which—in contrast to, e.g., FE${}^{2}$-approaches [39,45]—can only account for microstructural evolution within the limits of the model calibration. Since isotropic MAEs are considered, it is sufficient to describe the macroscopic behavior by the averaged principal stretches ${\bar{\lambda }}_{\alpha }^{\mathrm{iso}}$ and the three additional magneto-mechanical invariants

(17) $${\bar{I}}_{4}={\left|{\bar{H}}_{0}\right|}^{2}\;,\;{\bar{I}}_{5}={\bar{C}}^{-1}\;:\left({\bar{H}}_{0}{\bar{H}}_{0}\right)\;\mathrm{and}\;{\bar{I}}_{6}={\bar{C}}^{-2}\;:\left({\bar{H}}_{0}{\bar{H}}_{0}\right).$$

Choosing ${\bar{H}}_{0}$ as the independent magnetic quantity, the amended free energy for the macroscopic model can be expressed as $\bar{\Omega }=\bar{\Omega }\left({\bar{\lambda }}_{\beta }^{\mathrm{iso}},\bar{J},{\bar{I}}_{4},{\bar{I}}_{5},{\bar{I}}_{6}\right)$. Similar to the discussion in Section 2.1.2, $\bar{\Omega }$ can be divided into purely magnetic and mechanical parts—however, since coupling effects cannot be neglected on the macroscopic scale, such an approach requires $\bar{\Omega }$ to take the form:

(18) $$\bar{\Omega }={\bar{\Psi }}^{\mathrm{mech}}\left(\bar{C}\right)+{\bar{\Psi }}^{\mathrm{coup}}\left(\bar{C},{\bar{H}}_{0}\right)+{\bar{\Psi }}^{\mathrm{mag}}\left({\bar{H}}_{0}\right)-\frac{\mu_{0}}{2}\bar{J}{\bar{I}}_{5}.$$

Based on this, a possible model which is able to account for a strongly nonlinear behavior of the elastomer matrix as well as magnetic saturation effects of the particles is given by the following energetic contributions:

(19) $$\begin{array}{cc} {\bar{\Psi }}^{\mathrm{mech}} & =\sum_{p=1}^{N}\frac{{\bar{\mu }}_{p}}{{\bar{\alpha }}_{p}}\left(\sum_{\beta =1}^{N_{\lambda }}\nu_{\beta }{\bar{\lambda }}_{\beta }^{{\mathrm{iso}}^{{\bar{\alpha }}_{p}}}-3\right)+\frac{\bar{\kappa }}{4}\left({\bar{J}}^{2}-\ln{\bar{J}}^{2}-1\right), \end{array}$$

(20) $$\begin{array}{cc} \mu_{0}{\bar{\Psi }}^{\mathrm{coup}} & =-\frac{{\bar{\gamma }}_{1}}{{\bar{\delta }}_{1}}\ln\left[\cosh\left(\mu_{0}{\bar{\delta }}_{1}\sqrt{{\bar{I}}_{5}}\right)\right]+{\bar{\gamma }}_{2}\ln\left(1+\mu_{0}^{2}{\bar{\delta }}_{2}{\bar{I}}_{6}\right)+\frac{{\bar{\gamma }}_{3}\left(\bar{J}-1\right)}{2}\tanh^{2}\left(\mu_{0}{\bar{\delta }}_{3}\sqrt{{\bar{I}}_{5}}\right), \end{array}$$

(21) $$\begin{array}{cc} \mu_{0}{\bar{\Psi }}^{\mathrm{mag}} & ={\bar{\gamma }}_{4}\ln\left(1+\mu_{0}^{2}{\bar{\delta }}_{4}{\bar{I}}_{4}\right). \end{array}$$

For the fitting of the sets of macroscopic material parameters $\left\{{\bar{\mu }}_{p},\;{\bar{\alpha }}_{p},\;{\bar{\gamma }}_{k},\;{\bar{\delta }}_{k},\;\bar{\kappa }\right\}$, the systematic procedure described in [42,68] is applied: a total number of nine calibration and five validation load cases is performed with RVEs comprising 400 randomly distributed particles at different particle-volume fractions. To prevent an unsystematic RVE behavior, the minimum particle distance during the RVE geometry generation using a random sequential adsorption algorithm is set to 5% of the particle diameter. For the microscale simulations, the constitutive models according to Equations (11a) and (12a) were used—for the values of the individual parameters and the quality of the model calibration, the authors refer to [42,68].

**Figure 5:**
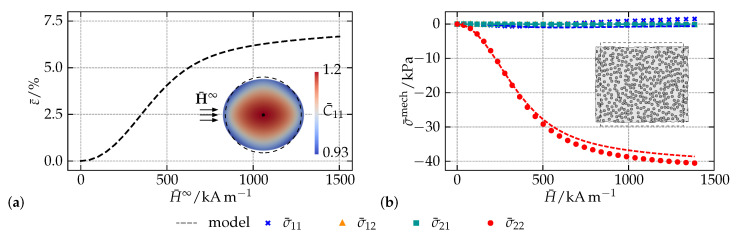
Magneto-deformation of a circular MAE sample with ϕ = 30% particle-volume fraction: (**a**) predicted magnetically induced deformation of the sample and contour plot of the macroscopic deformation field, and (**b**) comparison of the calibrated macro- and averaged micro-contnuum models regarding their predictions for the macroscopic mechanical stress at the center of the sample. Data taken from [74].

To demonstrate the model capabilities, the magneto-deformation of a circular macroscopic sample with a particle-volume fraction ϕ = 30% is investigated. In order to mimic the situation of a homogeneous far field, the sample is embedded into a sufficiently sized free space with negligible mechanical properties and the magnetic properties of free space, i.e., ${\bar{\mu }}_{r}=1$. Regarding the prediction of the magnetically induced strain $\bar{\varepsilon }=\Delta \bar{l}/{\bar{l}}_{0}$ given in Figure 5a, an initially quadratic behavior which saturates for large external fields ${\bar{H}}^{\infty }$ is observable. As already shown in the previous example, this response is typical for MAEs and qualitatively coincides with experimental findings. A closer look on the embedded contour plot of the sample reveals a highly inhomogeneous macroscopic deformation field $\bar{C}$: it results from the jump of the magnetic quantities on the MAE surface and illustrates the specific difficulties of experimental investigations on MAEs. Even macroscopic samples with a shape that allows for a homogenous magnetization cannot provide the basis for a shape independent material behavior.

In order to show the quality of the calibrated model, a localization step is performed in Figure 5b: for the center point of the MAE sample, the predictions of the macroscopic model with regard to the mechanical stress are compared to the averaged results of a microscopic simulation with an RVE that is subjected to the same macroscopic fields ${\bar{H}}_{0}$ and $\bar{F}$. The high accuracy of the macro-continuum model over the whole range of applied magnetic fields not only documents that it implicitly captures microstructural information, but also shows that the proposed framework represents an appropriate multiscale scheme which is a computationally efficient alternative to conventional FE${}^{2}$-approaches.

### 2.2. Dipole Approach to Particle Interactions

To capture the leading effects of long- and short-ranged magnetomechanical coupling responsible for the effective behavior of MAEs in an applied magnetic field, several approximations are introduced. The major simplification consists of the dipole approximation. In this framework, the magnetic interactions among filler particles are reduced to mutual dipole-dipole interactions. Thus, instead of resolving the, in general inhomogeneous, magnetization field in the volume of each individual inclusion, the problem is simplified to a single relation between the particles’ center positions. This reduces the computational effort considerably and allows for an approximate description of magnetic interactions among very large numbers of particles.

#### 2.2.1. Effective Macroscopic Behavior—Minimum of Global Energy

An adequate description of field-induced changes in the shape and mechanical properties of polymer networks with embedded magnetizable microparticles represents quite a challenging problem, even in the framework of the dipole approach to particle interactions. It is relatively easy to consider the influence of particle distribution on static properties [71,75] and dynamic moduli [76,77], using different structures defined on infinite lattices. However, the initial shape of the sample is proved to have a comparable and even a predominant effect on the change in shape [4,29], for example in MAEs with an isotropic distribution of the particles. The close correlation between the local particle structure on the microscale and the global geometric shape of the sample on the macroscale is already apparent from the magnetostatic laws alone. This fundamental interrelation of short-ranged structure effect and long-ranged shape effect makes it necessary not only to be able to describe both effects independently of each other, but also to combine them fundamentally in a unified approach in order to achieve a comprehensive characterization of MAEs.

Such unified approach in the context of the dipole approximation has been originally developed for linearly magnetizable particles placed on the sites of different lattices [29]. In that study, the effective magneto-mechanical behavior has been predicted by minimizing the free energy functional:

(22) $$U=U_{\mathrm{el}}+U_{\mathrm{mag}}$$

where $U_{\mathrm{el}}$ is the elastic energy of a deformed MAE sample due to the entropic elasticity of polymer chains and $U_{\mathrm{mag}}$ arises from the dipole-dipole interactions between magnetizable particles placed in an external magnetic field. This field is assumed to be homogeneous and hence direct interactions of the particles with the field may be skipped as they do not contribute to the minimization procedure. The elastic contribution $U_{\mathrm{el}}$ represents the effective mechanical properties of the composite in the absence of any magnetic fields, and it is usually implemented via a neo-Hookean material model. In the linear magnetization regime, i.e., M=χH with the magnetic susceptibility χ, the magnetic energy can be represented in a rather concise form [29]:

(23) $$U_{\mathrm{mag}}=-\frac{\mu_{0}}{2}\frac{\phi {H^{\infty }}^{2}}{\left(\chi^{-1}+n-\phi \left(f_{\mathrm{macro}}+f_{\mathrm{micro}}\right)\right)}.$$

The strength of the applied magnetic field is denoted by $H^{\infty }$. In the above equation, two scalar parameters $f_{\mathrm{micro}}$ and $f_{\mathrm{macro}}$ are introduced, with the first one quantifying the short range structure effect in regular, i.e., lattice, structures, and the second one the long-range shape effect. Note that these terms appear in the denominator of Equation (23), together with χ and demagnetizing factor n of a single particle, due to a self-consistent treatment of magnetic interactions. Minimizing Equation (22) for different lattice structures (simple cubic, body-centered cubic, hexagonal close-packed), it was found that the magneto-mechanical behavior very sensitively depends on the particular choice of lattice parameters upon introducing unrealistically strong long-range ordering among the particles.

**Figure 6:**
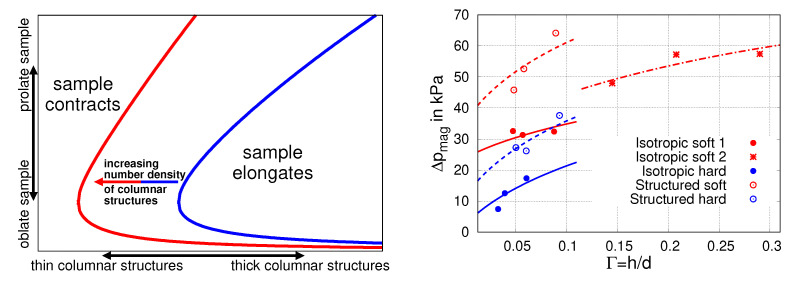
**Left side**: Part of a phase diagram as obtained in [32]. Whether an MAE sample contracts or elongates in direction of the applied magnetic field crucially depends on the sample form (oblate or prolate), the thickness of the columnar microstructure and number density of such structures in the sample, i.e., the overall amount of magnetizable particles. **Right side**: Systematic comparison between theory and experiment allowed for identifying the contribution from macroscopic sample shape and microscopic particle structure to the magnetically induced stress in confined MAE samples. Reproduced from Ref. [78] with permission from the Royal Society of Chemistry.

Apparently, presuming a perfect ordering which is intrinsic in the lattice structures may lead to undesirable artifacts. This problem can be eliminated by complementing the unified approach with a completely different characterization of particle distributions. An adequate description of practically relevant microstructures has been achieved in the mean-field version of the unified approach [32] via introduction of a continually varying density field, both for stochastically isotropic and elongated microstructures. The methodology developed for this purpose allows in part analytical solutions, on the basis of which the behavior of linearly magnetized spheroidal MAE samples was estimated over large parameter spaces and a wide range of situations. This allowed to draw comprehensive phase diagrams of the deformation behavior of MAE samples with elongated, or columnar, microstructures. On the left side of Figure 6, we schematically illustrate the results. Additionally, a discontinuous shape change for very oblate samples has been predicted, see Figure 10 from [32]. For the deformation behavior of samples with stochastically isotropic particle distribution, a good agreement with more detailed 2D continuum-mechanical simulation calculations was recently demonstrated [49].

Earlier theoretical considerations in the framework of the dipolar approach were restricted to the assumption of affine deformation on local scales [29,32]. Non-affine displacement of magnetic particles due to mutual hindrance of neighboring particles and its effect on the magneto-induced deformation was analyzed in a general study [79]. Allowing the particles to rearrange in the presence of magnetic field, the enhancement of the magneto-induced deformation in linearly magnetized spheroidal samples has been predicted. The unified approach has been further developed in order to calculate and analyze the interplay between short-ranged structure effect and long-ranged shape effect for the nonlinearly magnetized samples of realistic shapes (cylinder, cuboids). In such generalized formulation, the governing equations turn into tensorial forms [78]. In particular, it was shown that the average magnetization $\langle M\rangle$ in the axially symmetric samples, with the external field applied along the axis of symmetry, can be compactly expressed in scalar form as [78]:

(24) $$\langle M\rangle =M\left(H^{\infty }+\left(\phi \langle f_{\mathrm{macro}}\rangle +\langle f_{\mathrm{micro}}\rangle -n\right)\langle M\rangle \right)$$

Here, M denotes the magnetization function, see Equation (1), $\langle f_{\mathrm{macro}}\rangle$ defines the demagnetizing factor of a sample along its axis of symmetry and $\langle f_{\mathrm{micro}}\rangle$ describes the actual, in general arbitrary or irregular, microstructure. This theoretical development allowed for quantifying the contribution of microstructure effect into the magnetically induced stress in confined MAE samples, as described in detail in [50,78]. Clear trends for the isotropic and structured samples have been established by fitting the theory predictions, see the right side of Figure 6, to the measured stress data in a specially designed experiment [78].

The presence of external magnetic field transforms initially isotropic MAE samples into transversely isotropic ones with an axis of symmetry defined by the direction of magnetic field. Uniaxial deformations applied along and perpendicular to the field direction lead to a strong anisotropy in the magnetically induced stress response, as predicted in a very recent work [80]. In this study, an attempt was made to derive an effective material model from the free energy functional based on the dipole approximation for magnetic interactions. Importantly, a strong magneto-mechanical coupling between the internal magnetic field and the sample shape is treated self-consistently from the very beginning, being a direct outcome of the minimization of free energy (Equation (22)). This is in contrast to the phenomenological models, in which the form of this coupling is postulated through introduction of multiple pseudo-invariants.

The unified approach is based on the minimization of free energy and is therefore restricted to description of static magneto-mechanical behavior. Moreover, the magneto-induced changes in the microstructure can be captured only on average by considering an evolution of a single scalar parameter $f_{\mathrm{micro}}$. To be able to describe these changes in more detail, we also considered an alternative approach to the magnetic and elastic interactions, as will be described in the next subsection.

#### 2.2.2. Explicit Particle Structures—Balance of Local Forces

Naturally, the energetic minimum of Equation (22), yielding the equilibrium state, corresponds to the balance of magnetic and elastic forces. However, to obtain analytic relations directly from the energy based approach, additional constraints were introduced. For example, in the case of affine elastic coupling to a uniaxial macroscopic deformation, it is possible to treat the ’collective’ repositioning in terms of only one single deformation parameter, i.e., the relative length change of the sample in direction of applied field, $\varepsilon =\frac{\Delta L}{L}$. In order to account explicitly for the local restructuring under the action of an applied field, it is more convenient to calculate the mutual magnetic forces as driving forces for the particle rearrangements. Thus, as an alternative to the effective energy approach discussed before, the magneto-mechanical problem can be formulated equivalently in terms of magnetic and elastic forces acting on the individual filler particles. In the literature, different models based on mutual dipole-dipole forces in bead spring or continuum approaches have been introduced to simulate the behavior of MAEs [81,82,83,84,85]. In the following, a force-based formulation for a sample of filler particles will be specified in close correspondence to the preceding subsection.

In principal, the magnetic forces are obtained as the negative gradient field of the magnetic energy. Accordingly, the starting point here is the general equation for the magnetic energy of a material/sample of volume $V_{s}$ in some external field $H^{\infty }$ [32,86]:

(25) $$U_{\mathrm{mag}}=-\mu_{0}\int_{V_{s}}\;d^{3}r\left\{\int_{0}^{H(r)}\;\;M\left(r\right)\;\cdot \;dH\;-\;\frac{1}{2}M\left(r\right)\;\cdot \;\left(H\left(r\right)-H^{\infty }\right)\right\}\;.$$

In case of an MAE, representing a composite of non-magnetizable matrix, where M=0, with *N* embedded magnetic/magnetizable inclusions, the magnetic energy becomes a sum of integrals over the corresponding inclusion volumes. The relation between magnetization M and local magnetic field H is given via Equation (1).

In the dipole approach, we assign each particle, labeled i∈N, an individual dipole moment $m_{i}$ located at its center position. This dipole moment represents the average magnetization of the inclusion:

(26) $$m_{i}=\int_{v_{i}}d^{3}r\;M\left(r\right)=v_{i}{\langle M\rangle }_{i}.$$

Here, $v_{i}$ denotes the volume of particle *i* and the average ${\langle \;\cdot \;\rangle }_{i}$ in the r.h.s. means that it has taken over this volume $v_{i}$. Assuming a homogeneous magnetic field over $v_{i}$, and thus also a homogeneous magnetization of the sphere, the dipole field exactly describes the magnetic field generated by the particle in its surrounding.

The dipole approximation is equivalent to the assumption that magnetic field H(r) can be considered as constant over the extent of each particle. Since the external field is usually considered homogeneous, or will not change notably on the range of micron-sized particles anyways, the error of the dipole approximation is essentially due to inhomogeneities of the demagnetization fields among neighboring particles. It has been shown [27,86,87] that the dipole approximation model starts to deviate from exact calculations only as particles come closer than $∼1.5\;d_{p}$ to each other, where $d_{p}$ denotes a particle diameter.

In the following, center positions of the particles are denoted by $r_{i}$ and we introduce the short notation $X_{i}=X\left(r_{i}\right)$. Then, applying the dipole approximation the volume integral in Equation (25) can be performed straightforwardly over the volume of each inclusion. The result is a sum over all *N* dipoles:

(27) $$U_{\mathrm{mag}}=-\mu_{0}\sum_{i}^{N}\left\{\int_{0}^{H_{i}}\;m_{i}\;dH\;-\;\frac{1}{2}\left(m_{i}H_{i}-m_{i}\;\cdot \;H^{\infty }\right)\right\}.$$

Note, due to Equation (1), we immediately find $m_{i}\;\|\;H_{i}$, whereas $H^{\infty }$ is not necessarily aligned with the dipole moments. In the dipole approximation, Equation (1) becomes:

(28) $$\frac{m_{i}}{v_{i}}=M\left(H_{i}\right)\frac{H_{i}}{\left|H_{i}\right|},$$

For a sample with given macroscopic shape and particle distribution, we obtain from Equation (27) the magnetic contribution to the total energy. Note that the application of Equation (27) requires the self-consistent solution of a set of 3N coupled equations of the form of Equation (28). As outlined in Section 2.2.1, the energetic description is useful to calculate the equilibrium state and also serves as the basis for an effective material model describing for example finite deformation processes.

It was shown explicitly [87] for two equally sized particles with a linear magnetization behavior, i.e., M(H)=χH, that the magnetic forces obtained as the gradient field of the magnetic energy in Equation (27) are identical to the well-known dipole-dipole interaction forces. In Appendix A, we demonstrate the validity of this identity for any number of differently sized particles assuming an arbitrary magnetization function M(H) in Equation (1). In the dipole approximation, the magnetic force $f_{k}^{d}$ acting on some particle *k* in a sample of *N* magnetized inclusions is given as:

(29) $$f_{k}^{d}=-\frac{3\mu_{0}}{4\pi }\sum_{i\neq k}^{N}\left\{\frac{r_{ki}^{2}\left(\left(r_{ki}\;\cdot \;m_{k}\right)m_{i}+\left(r_{ki}\;\cdot \;m_{i}\right)m_{k}+\left(m_{i}\;\cdot \;m_{k}\right)r_{ki}\right)-5\left(r_{ki}\;\cdot \;m_{i}\right)\left(r_{ki}\;\cdot \;m_{k}\right)r_{ki}}{r_{ki}^{7}}\right\}.$$

Here, $r_{ki}=r_{i}-r_{k}$ denotes the vector connecting particles *k* and *i* and $r_{ki}=\left|r_{ki}\right|$. Once the magnetization, i.e., the magnetic dipoles $m_{i}$, of the particles are specified, the magnetic forces acting on each of them are also known, and the result is consistent to the energy based approach.

The local field $H_{i}$ is the sum of the external field, the (self-)demagnetization field and the dipole fields of all the other particles:

(30) $$H_{i}=H^{\infty }-n\frac{m_{i}}{v_{i}}+\frac{1}{4\pi }\sum_{j\neq i}^{N}\frac{3\left(m_{j}\;\cdot \;r_{ji}\right)r_{ji}-r_{ji}^{2}m_{j}}{r_{ji}^{5}}.$$

Consequently, to obtain the individual $m_{i}$, we require the self-consistent solution of a 3N system of coupled nonlinear equations as formulated through Equations (28) and (30). This calculation is performed numerically via classical Newton-Raphson technique providing a fast and stable convergence after only few iteration steps.

In the energetic formulation, we usually implement a macroscopic mechanical model to describe the elastic properties of the sample. That is, we introduce effective moduli of the composite material in the absence of magnetic fields, i.e., for the filler reinforced matrix at given volume fraction of embedded hard particles. In contrast, to account for particle rearrangements on local scale we require a relation between the displacement $\Delta r_{i}$ of some individual particle and the set of applied forces $\left\{f_{k},\;k\in N\right\}$ that are acting on the inclusions. The embedding medium shall thereby be characterized by its ’pure’ elastic modulus, containing a given distribution of surrounding hard particles. Using linear elasticity theory, Puljiz et.al. obtained an analytic expression considering in first approximation mutual 2- and 3-body elastic interactions among spherical particles [30,88]. In our present work, we assume an isotropic and incompressible elastic medium. Accordingly, we set ν=0.5 and 3μ=E, where ν denotes the Poisson ratio, μ the shear modulus, and *E* the elastic modulus. From [88], we then find for each particle in the sample a linear dependency of the form:

(31) $$r_{i}-r_{0_{i}}=\Delta r_{i}=\frac{1}{8\pi Ed_{p}}\sum_{j}^{N}{\hat{D}}_{ij}\;\cdot \;f_{j}.$$

Note that subscript ${\;}_{0}$ refers to the initial particle positions in the absence of any forces, i.e., in the absence of magnetic fields, and $f_{j}$ denotes the force acting on the spherical inclusion *j*. The so defined tensors ${\hat{D}}_{ij}$ are dimensionless and read for i≠j:

(32) $${\hat{D}}_{ij}=\left(\frac{3d_{p}}{r_{0_{ij}}}+\frac{d_{p}^{3}}{2r_{0_{ij}}^{3}}\right)\hat{I}+\left(\frac{3d_{p}}{r_{0_{ij}}^{3}}-\frac{3d_{p}^{3}}{2r_{0_{ij}}^{5}}\right)r_{0_{ij}}r_{0_{ij}}+\frac{15d_{p}^{4}}{16}\sum_{k\neq i,j}^{N}\left(1-3{\left(\frac{r_{0_{ik}}\;\cdot \;r_{0_{jk}}}{r_{0_{ik}}r_{0_{jk}}}\right)}^{2}\right)\frac{r_{0_{ik}}r_{0_{jk}}}{r_{0_{ik}}^{3}r_{0_{jk}}^{3}},$$

and for i=j:

(33) $${\hat{D}}_{ii}=8\hat{I}-\frac{15d_{p}^{4}}{8}\sum_{k\neq i}^{N}\frac{r_{0_{ik}}r_{0_{ik}}}{r_{0_{ik}}^{6}}.$$

Here, $\hat{I}$ represents the identity tensor. We denote the dyadic product of two vectors a and b in the form ab, whereas a·b implies the scalar product. The first term in Equation (33) describes the single particle contribution due to an elastic medium without any other inclusions. The last term in Equation (32) accounts for 3-body hydrodynamic interactions and the rest in Equations (32) and (33) refers to corresponding 2-body interactions.

Thus, the final equilibrium positions $r_{i}=r_{0_{i}}+\Delta r_{i}$ when applying some homogeneous $H^{\infty }$ are found upon insertion of Equation (29) in Equation (31) assuring at the same time the self-consistency of the magnetic dipoles of all particles with respect to these new positions via Equations (30) and (28). The numerical, or computational, effort for the calculation increases with order $O({\left(3N\right)}^{3})$ and becomes quite cumbersome for large systems (N≫1). The *bottleneck* in the computation arises from the 3-particle hydrodynamic interactions, i.e., the last term in Equation (32). However, due to its reference to initial positions, the contribution for each ${\hat{D}}_{ij}$ only needs to be specified once for a given system. Up to N=1000 particles, the solution of this algorithm is moderately accessible ($O(10^{2}\;\mathrm{seconds})$). To test the accuracy of the present approximation approach, we will consider finite samples with few embedded particles in Section 3 and compare the results to the refined continuum description.

**Figure 7:**
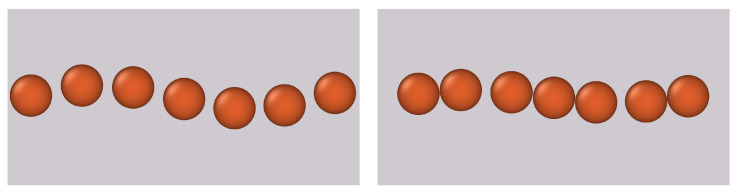
Snapshots of the dynamical simulation model presented in Appendix B. We consider an incompressible matrix with elastic modulus of E=40 kPa and a magnetization behavior of the particles following Equations (1) and (13a). Left side: Initial configuration in the form of a regular helical chain before applying an external magnetic field. Right side: The final configuration when applying a field of $B^{\infty }=1T$ along the main chain axis. The full video can be found in the Supplementary Materials (Video2.avi). The snapshots and videos have been visualized using OVITO [89].

The above defined algorithm provides a static process from initial particle positions $r_{0_{i}}$ (the prepared equilibrium in the absence of magnetic fields) to final positions $r_{i}$ (the equilibrium in the presence of magnetic fields). Alternatively, in Appendix B, we suggest a simulation model based on the force balancing description of the problem. This extension follows in a straightforward manner upon insertion of corresponding forces into the fundamental Newtons equation and thereby allows for including dynamical aspects of particle repositioning. Furthermore, in contrast to the micro-continuum approach and the static force balancing approach, the dynamical simulation model can naturally prevent the overlap or collapse of neighboring particles in very soft matrices upon introducing a hard sphere repulsion term (see Appendix B). In Figure 7, we display two snapshots of the dynamical simulation for particle rearrangement. The matrix is chosen to be so soft that neighboring particles would collapse into each other due to predominant magnetic forces over elastic ones. In the dynamical simulation model, the particles stick to form pairs/clusters; see the right side in Figure 7. Under such conditions, both aforementioned approaches would fail. As long as the elastic matrix is stiff enough to prevent such collapsing events, the final equilibrium positions of the particles when applying an external magnetic field are identical to the static force balancing approach. Example videos of rearrangement processes, as calculated via the suggested simulation model specified in Appendix B, are attached in the Supplementary Materials.

## 3. Comparison of the Approaches

In this section, the presented micro-modeling strategies are compared with regard to their predictions for the magneto-deformation of MAE samples with a specific particle distribution. Since chain-like microstructures are of significant interest in both experimental [8,90,91] and theoretical [34,79,92,93] investigations, idealized samples comprising helical chains with different angles between two neighboring particles are analyzed. Such structures represent the three-dimensional generalization of *wavy* chains, which, in the simplified two-dimensional case, have been found to be a possible microstructural explanation for the often observed positive magneto-deformation of MAEs with chain-like microstructures [94]. However, recent findings of the authors [56,79] show that two-dimensional analyses tend to overestimate the resulting effects in MAEs and experimentally observed effects cannot be explained with interactions in a simplified wavy microstructure. To this end, the subsequent investigation not only compares the two micro-modeling strategies but can also help to understand why some particle arrangements are more prone to positive magneto-deformation than others and serve as a basis for studies on more realistic, randomized chain-like structures.

**Figure 8:**
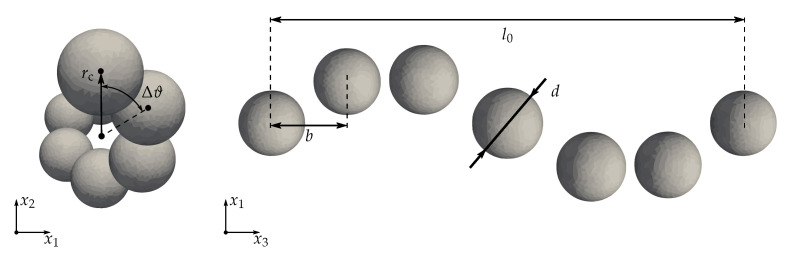
Schematic illustration of helical structures: front- and bottom-views with introduction of relevant geometrical parameters. The depicted structure represents a full helical chain with $\Delta \vartheta =60{}^{\circ }$, b=6μm, d=5μm and $\alpha =\frac{r_{c}}{b}=0.6$.

Within this study, helical chains with angles $\Delta \vartheta =\{45{}^{\circ },\;60{}^{\circ },\;90{}^{\circ },\;180{}^{\circ }\}$ between adjacent particles are embedded into an elastomer surrounding which has a size that is sufficient to apply a homogeneous far field $B^{\infty }$ in the chain axis direction with $B^{\infty }=1T$. For every type of helical chains, a parameter $\alpha =\frac{r_{c}}{b}$, with *b* being the particle distance in the chain axis direction, controls the chain radius $r_{c}\;$, i.e., the distance of the particle centers to the chain axis. This allows for investigating helical structures with different distance ratios, see Figure 8 for an illustration of the chain geometry, and represents a continuation of the studies performed in [56].

For the numerical simulations, a modulus $E^{m}=200\;k\mathrm{Pa}$ is assumed for the quasi-incompressible matrix ($\nu^{m}=0.49$ within the FE simulations of the micro-continuum approach), while the magnetization function of the particles is characterized by $M_{s}=841\;kA/m$ and $\zeta =2.18\times 10^{-2}\;m/\mathrm{kA}$ according to Equation (13a). In order to quantify the magneto-deformation ε, the relative change of the structure length *l* is compared to the length $l_{0}$ in the absence of magnetic fields, i.e., $\varepsilon =\frac{\Delta l}{l_{0}}$. The diameter and the distance of the particles in the chain axis direction are fixed, d=5μm and b=6μm, and all types of helical structures are analyzed for a number of $n_{p}\in \left\{3,\cdots ,17\right\}$ particles to allow an investigation of up to 2 full chain cycles within the elastomer surrounding.

**Figure 9:**
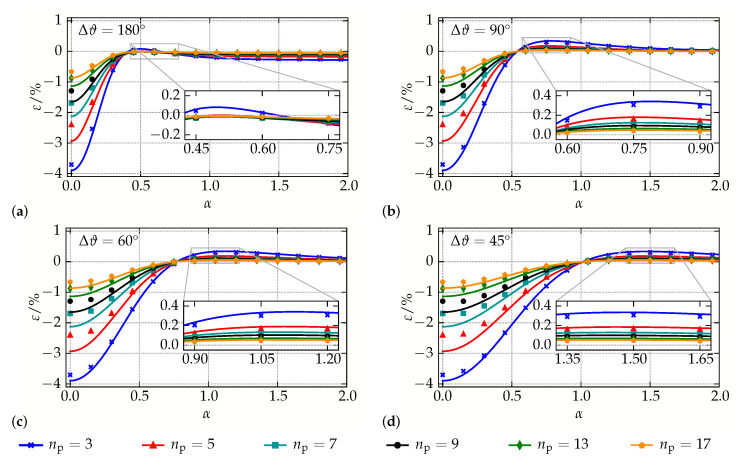
Results for helical chains with varying angle between adjacent particles: magneto-deformation plotted over the distance ratio α for (**a**) $\Delta \vartheta =180{}^{\circ }$, (**b**) $\Delta \vartheta =90{}^{\circ }$, (**c**) $\Delta \vartheta =60{}^{\circ }$, and (**d**) $\Delta \vartheta =45{}^{\circ }$.

In Figure 9, the results of the comparison are summarized for all helical chains. Within the individual subplots, data obtained with micro-continuum simulations are indicated by markers, whereas the results of the particle-interaction model are indicated by solid lines of the same color. Note that all results represent bare findings of the comparison of the two modeling approaches, i.e., they are obtained by just applying the same magnetic field $H^{\infty }$ and material parameters but entail no further fitting or adjustment.

Starting with the widely investigated plane chains with $\Delta \vartheta =180{}^{\circ }$ in Figure 9a, three main conclusions can be drawn. First of all, the comparison of the micro-continuum and particle-interaction models shows a remarkable agreement over almost the whole range of analyzed distance ratios α: only for very small values of α, i.e., for the case of almost straight chains, a difference between the two approaches is visible. Keeping in mind that, with b/d=1.2, adjacent particles are very close to each other for small values of α; these discrepancies can be ascribed to errors caused by the dipole approximation and might also be related to the assumption of a linear elastic matrix material within the particle-interaction model. A second finding is that both approaches consistently predict a contraction of wavy chains for almost all distance ratios α. Only in a very small range 0.4≤α≤0.65 a positive magneto-deformation is observed, see the magnified inset shown in Figure 9a. Finally, it is apparent that the effect of magneto-deformation decreases with the number of particles $n_{p}$ considered within the models. While the particle interactions in short structures yield a strong effect, especially for almost straight chains, this interaction is reduced systematically if $n_{p}$ is increased. This is a clear evidence for boundary effects which dominate the behavior of short chains and are of minor importance if larger, elongated structures are considered. Altogether, the results for the analyzed geometries are in line with other studies [56] and point out that the experimentally observed behavior of MAEs with chain-like microstructures cannot be explained with oversimplified wavy chains.

Since helical chains with $\Delta \vartheta =180{}^{\circ }$ only represent a plane problem in which the complex interactions of particles in MAEs cannot be captured, the analysis is expanded to more elaborate helices with $\Delta \vartheta =\{90{}^{\circ },60{}^{\circ },45{}^{\circ }\}$, as can be seen in Figure 9b–d. Again, the consistency of the results obtained with the two different modeling approaches is striking: within all examples, they only differ for very small values of α. Comparable to the preceding example, all helices show a negative magneto-deformation if the distance ratio is small, i.e., the chains are almost straight. Interestingly, the transition from contraction to elongation is shifted towards higher values of α, if Δϑ decreases. Additionally, it can be seen that—independent of the number of particles within the chains—their higher complexity allows for finding positive magneto-deformation within a broad range of α. In contrast to the helices with $\Delta \vartheta =180{}^{\circ }$, there is no second transition from elongation to contraction for larger values of α. A closer look at the magnified insets in Figure 9b–d reveals that the maximum elongations are almost independent of Δϑ. Again, boundary effects lead to a much stronger magneto-deformation, especially if the analyzed geometry does not represent at least a full chain cycle. However, even if up to two full cycles are considered, an elongation of the structure is still found.

In summary, the present study shows that more complex helical structures represent an adequate generalization of the well-investigated wavy chains: their complexity allows for a broader range of possible effects and supports the idea that the particle arrangement in chain-like structures can trigger a transition from contraction to elongation in an applied magnetic field. In contrast to previous works, the results of this study show that such a transition cannot be realized with wavy chains: to attain a positive magneto-deformation with simplified chain-like structures, the particles must be arranged in a helical shape with $\Delta \vartheta \neq 180{}^{\circ }$. As the primary focus of the analysis is a systematic comparison of the micro-continuum and particle-interaction models presented in Section 2, the remarkable agreement of the results obtained with both approaches must again be emphasized. This finding is in line with the outcome of a former comparison of the approaches for the simplified two-dimensional case [49] and promotes the idea of a hybrid multiscale framework for the analysis of realistic MAE samples in which computationally expensive FE simulations can be replaced by the less costly particle-interaction approach.

## 4. Conclusions

Within this work, two different modeling strategies for magneto-active elastomers are presented and compared with regard to their predictions for the magneto-deformation of chain-like helical structures in an elastomer surrounding. After both modeling strategies are introduced and their applications are illustrated by means of representative examples, the problem of particle interactions in elongated microstructures is analyzed. The investigation of helical chains with different angles Δϑ between adjacent particles represents a consistent continuation of former studies and allows for identifying effects of local particle rearrangements in MAEs on their macroscopic behavior. In line with a former comparison of the presented modeling approaches, the results show a remarkable agreement for all structures under investigation. Only in situations where basic assumptions of the particle-interaction model lose their validity, small deviations are found. Regarding the influence of different particle arrangements on the magneto-deformation of the structure, complex helical chains with $\Delta \vartheta \neq 180{}^{\circ }$ are found to allow for contraction as well as elongation depending on the chain geometry.

Certainly, the obtained results cannot fully explain the magneto-mechanical coupling behavior in MAEs with chain-like microstructures, since it also—and in many situations predominantly—depends on macroscopic shape effects as well as the elastic properties of the matrix material, among others. However, the findings of this study show that, at least on the microstructural level, the chain geometry can have a significant influence on the coupling behavior. To this end, the results should be a basis for further studies on MAEs with chain-like microstructures and, moreover, emphasize again that the complex interactions in those materials cannot be explained by just considering planar *wavy* chains.

## Figures and Tables

**Figure A1:**
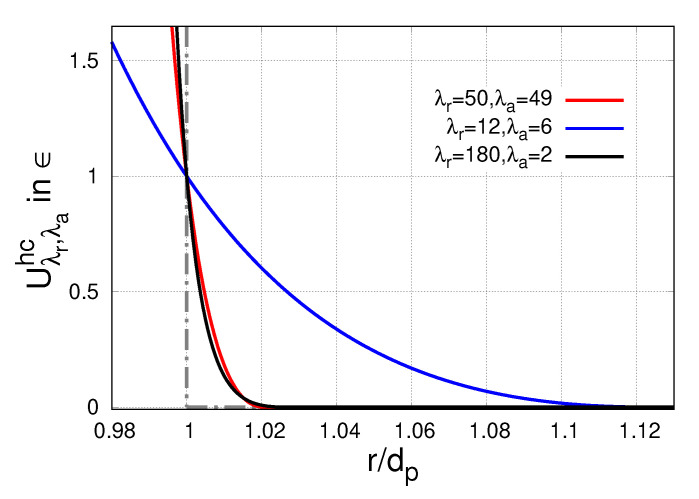
Plotting the generalized cut-and-shifted potentials from Equation (A15) for selected $\lambda_{r}$ and $\lambda_{a}$. As a guide to the eye, we additionally sketch the ideal hard core potential as a grey dashed-dotted line. The classical Lennard-Jones potential (blue) can only very poorly resemble the ideal form—especially, since neighboring microspheres should only start to ’feel’ each other (beside the hydrodynamic interaction included in $f^{\mathrm{el}}$ in Equation (A13)), as they really come to physical contact at $r\approx d_{p}$, making alternative forms like $U_{50,49}^{\mathrm{hc}}\left(r\right)$ or $U_{180,2}^{\mathrm{hc}}\left(r\right)$ much more reasonable.

## Data Availability

On inquiry, the data presented in this study is available from the authors.

## Supplementary Materials

The following are available online at https://www.mdpi.com/1996-1944/14/2/434/s1, Video S1: Video1.avi, Video S2: Video2.avi, Video S3: Video3.avi, Explanatory information S4: SuppInf.pdf.

## Appendix A

Here, for the sake of generality, we account for the possibility of a position-dependent external field $H^{\infty }$. To simplify the derivation, we will make use of the form of Equation (1), resp. Equation (28), where the magnetization, i.e., the dipole moment, is aligned with the local magnetic field $H_{i}$.

We start from Equation (27) and denote the position of particle *k* as $r_{k}$. Hence, the magnetic force on particle *k* is given via the negative gradient with respect to $r_{k}$:

(A1) $$f_{k}^{\mathrm{mag}}=-\nabla_{k}U_{\mathrm{mag}}=\mu_{0}\sum_{i}^{N}\left\{m_{i}\nabla_{k}H_{i}-\frac{\nabla_{k}}{2}\left(m_{i}H_{i}\right)+\frac{\nabla_{k}}{2}\left(m_{i}\;\cdot \;H_{i}^{\infty }\right)\right\}.$$

Note that the integral term in Equation (27) depends explicitly only on the local magnetic field $H_{i}$ up to which one has to integrate. Hence, we used the chain rule identity $\nabla_{k}M_{i}=\left(\nabla_{k}H_{i}\right)\frac{\partial M_{i}}{\partial H_{i}}$. Already at this step, one notes that the derivation is independent of the magnetization function M(H). From Equation (A1), follows:

(A2) $$f_{k}^{\mathrm{mag}}=\frac{\mu_{0}}{2}\sum_{i}^{N}\left\{m_{i}\nabla_{k}H_{i}-H_{i}\nabla_{k}m_{i}+\nabla_{k}\left(m_{i}\;\cdot \;H_{i}^{\infty }\right)\right\}.$$

immediately follows.

Although we have $m_{i}\|H_{i}$, one must be aware that $H_{i}^{\infty }$ is not necessarily aligned with the dipole moments. Therefore, we change to tensor notation and rearrange Equation (A2) further to:

(A3) $$f_{k}^{\mathrm{mag}}=\frac{\mu_{0}}{2}\sum_{i}^{N}\left\{\left(\nabla_{k}\left(H_{i}-H_{i}^{\infty }\right)\right)\;\cdot \;m_{i}-\left(\nabla_{k}m_{i}\right)\;\cdot \;\left(H_{i}-H_{i}^{\infty }\right)+2\left(\nabla_{k}H_{i}^{\infty }\right)\;\cdot \;m_{i}\right\}.$$

Note the expressions of the form $\nabla_{k}H$ represent tensors of rank 2. We use the operator ∇ in standard notation as $\nabla =e_{\alpha }\frac{\partial }{\partial r_{\alpha }}$, where $e_{\alpha }$ forms the basis vectors for example in Cartesian coordinates (α=x,y,z). In case of a constant external field, the last term in Equation (A3) vanishes, otherwise it only contributes if i=k and accordingly it represents the well-known force on a fixed magnetic dipole in varying external field (non-vanishing gradient of the field):

(A4) $$f_{k}^{\mathrm{ext}}=\mu_{0}\left(\nabla_{k}H_{k}^{\infty }\right)\;\cdot \;m_{k}.$$

Splitting the total magnetic force in Equation (A3) accordingly, we write:

(A5) $$f_{k}^{\mathrm{mag}}=f_{k}^{\mathrm{ext}}+f_{k}^{d}.$$

Here, $f_{k}^{d}$ contains the first two terms of Equation (A3) representing the dipole-dipole interaction force. In analogy to Equation (30), we find in the dipole approximation the following relation:

(A6) $$H_{i}-H_{i}^{\infty }=-n_{i}\frac{m_{i}}{v_{i}}+\frac{1}{4\pi }\sum_{j\neq i}^{N}m_{j}\;\cdot \;\frac{3r_{ji}r_{ji}-r_{ji}^{2}\hat{I}}{r_{ji}^{5}}.$$

Parameter $n_{i}$ denotes the demagnetization factor of particle *i*. For a spherical inclusion, we have $n_{i}=1/3\;,\;\forall i$, but, for generality, it is left as a parameter here. Note that in the case of ellipsoidal inclusions, the presented dipole approach is fully equivalent and works with the same accuracy as for spheres, only $n_{i}$ turns into the tensorial form to describe the demagnetization factor of an ellipsoid. The derivative of Equation (A6) with respect to the position of particle *k* gives:

(A7) $$\nabla_{k}\left(H_{i}-H_{i}^{\infty }\right)=-\frac{n_{i}}{v_{i}}\nabla_{k}m_{i}+\frac{1}{4\pi }\sum_{j\neq i}^{N}\left\{\frac{3r_{ji}r_{ji}-r_{ji}^{2}\hat{I}}{r_{ji}^{5}}\;\cdot \;\nabla_{k}m_{j}+m_{j}\;\cdot \;\nabla_{k}\left(\frac{3r_{ji}r_{ji}-r_{ji}^{2}\hat{I}}{r_{ji}^{5}}\right)\right\}.$$

Here, the last factor on the r.h.s. represents a tensor of rank 3. Using Equation (A7) and (A6) in Equation (A3), the result for the dipole-dipole contribution $f_{k}^{d}$ reads:

(A8) $$f_{k}^{d}=\frac{\mu_{0}}{2}\sum_{i}^{N}\left\{\frac{1}{4\pi }\sum_{j\neq i}^{N}m_{i}m_{j}:\nabla_{k}\left(\frac{3r_{ji}r_{ji}-r_{ji}^{2}\hat{I}}{r_{ji}^{5}}\right)\right\}.$$

Note that we used the notation “:” for the double dot product, also known as double inner product. Due to the symmetry i↔j and noticing that the application of the gradient $\nabla_{k}$ is only non-zero if either i=k or j=k, we further obtain:

(A9) $$f_{k}^{d}=\frac{\mu_{0}}{4\pi }\sum_{i\neq k}^{N}\left\{m_{i}m_{k}:\nabla_{k}\left(\frac{3r_{ki}r_{ki}-r_{ki}^{2}\hat{I}}{r_{ki}^{5}}\right)\right\}.$$

With $r_{ki}=r_{i}-r_{k}$, and accordingly $\nabla_{k}=-\nabla_{ki}$ (denoting the derivative with respect to $r_{ki}$), the application of the nabla operator yields:

(A10) $$f_{k}^{d}=-\frac{\mu_{0}}{4\pi }\sum_{i\neq k}^{N}\left\{m_{i}m_{k}:\frac{3r_{ki}^{2}\left(r_{ki}\hat{I}+\sum_{\alpha }e_{\alpha }r_{ki}e_{\alpha }+\hat{I}r_{ki}\right)-15r_{ki}r_{ki}r_{ki}}{r_{ki}^{7}}\right\}.$$

Finally, carrying out the double dot product, we find the well-known interaction force for mutually interacting dipoles:

(A11) $$f_{k}^{d}=-\frac{3\mu_{0}}{4\pi }\sum_{i\neq k}^{N}\left\{\frac{r_{ki}^{2}\left(\left(r_{ki}\;\cdot \;m_{k}\right)m_{i}+\left(r_{ki}\;\cdot \;m_{i}\right)m_{k}+\left(m_{i}\;\cdot \;m_{k}\right)r_{ki}\right)-5\left(r_{ki}\;\cdot \;m_{i}\right)\left(r_{ki}\;\cdot \;m_{k}\right)r_{ki}}{r_{ki}^{7}}\right\}.$$

Thus, independent of the magnetization behavior, particle sizes, and particle demagnetization factors, the dipole-dipole interaction in Equation (A11) determines the force acting on an individual particle due to the presence of the surrounding inclusions in the first order dipole approximation as formulated in Equation (26) and (28). The form of Equation (A11) is rewritten in Equation (29) in the main text.

## Appendix B

The starting point to set up a simulation model for particle rearrangement processes in MAEs is Newton’s equation:

(A12) $$f_{i}^{d}+f_{i}^{\mathrm{el}}+f_{i}^{\mathrm{hc}}+f_{i}^{\mathrm{th}}+f_{i}^{\mathrm{fr}}=m_{i}{\ddot{r}}_{i}.$$

The left-hand side represents the sum of all forces acting on some particle *i* at some time *t*. We denote $m_{i}$ as the particle mass and ${\ddot{r}}_{i}$ as the instantaneous acceleration. For monodisperse systems, clearly $m_{i}=m,\;\forall i$. In the following, we discuss the meaning and the implementation of each term in the l.h.s. of Equation (A12) for the case of micron-sized magnetizable particles embedded in an incompressible linear elastic medium.

The magnetic force $f_{i}^{d}$ acting on any particle *i* is again described via the dipole model Equation (29) where we assure self-consistency of the magnetic moments through Equations (28) and (30).

Contribution $f_{i}^{\mathrm{el}}$ shall provide the ’restoring’ force due to the elastic matrix when the particles change their positions ($\left\{r_{0_{j}}\right\}\to \left\{r_{j}\right\}$). To implement $f_{i}^{\mathrm{el}}$, we make use of the approximate results from the linear elasticity model as obtained by Puljiz et al. [30,88]. For an incompressible, isotropic matrix material, Equation (31) provides the superimposed relations between the set of locally applied forces $\left\{f_{j}\right\}$ and the set of displacements $\left\{\Delta r_{j}\right\}$ including hydrodynamic 2- and 3-body interactions. These linear relations must be apparently unique, i.e., the displacements $\Delta r_{j}$ can only become zero for all *j* simultaneously if, and only if, no forces are applied at all, $f_{j}=0\;\forall j$, as otherwise the elastostatic model is ill-defined. Thus, likewise, as a set of forces provides the corresponding set of displacements, also a given set of displacements $\left\{\Delta r_{j},\;j\in \left[1,N\right]\right\}$ provides the corresponding set of forces that must be applied to each inclusion. Accordingly, since Equation (31) determines the ’new’ elastically equilibrated positions with respect to the force free situation $\left\{r_{0_{j}},\;j\in \left[1,N\right]\right\}$, we know that the elastic restore forces upon repositioning are determined as:

(A13) $$f_{i}^{\mathrm{el}}=-f_{i}=-8k\sum_{j=1}^{N}{\hat{K}}_{ij}\;\cdot \;\Delta r_{j}.$$

Here, we defined the elasticity prefactor $k:=\pi Ed_{p}$. The dimensionless tensors ${\hat{K}}_{ij}$ are the entries i,j of that matrix of tensors found upon inversion of the matrix of tensors ${\left[D\right]}_{ij}={\hat{D}}_{ij}$. i.e., besides the minus sign in Equation (A13), it represents the inversion of Equation (31).

Since under general conditions the elastic restore forces $f_{i}^{\mathrm{el}}$ can not prevent neighboring particles from overlapping, or even merge into each other due to the singularity of the dipole-dipole force as $r_{ij}\to 0$ [73], the hard core repulsion $f_{i}^{\mathrm{hc}}$ prevents such artifactual events from happening. We will choose a centrosymmetric, short-ranged repulsive potential of the type of a generalized Lennard-Jones potential. Such generalized potentials have been introduced long ago [95] and are known as Mie potentials:

(A14) $$V_{\mathrm{Mie}}\left(r\right)=\frac{a}{r^{\nu }}-\frac{b}{r^{\mu }}.$$

Originally, such potentials have been introduced to characterize inter-atomic, or inter- and intra-molecular, interactions, where *r* denotes the distance between the center positions of atoms, resp. molecules. Equation (A14) contains the well-known Lennard-Jones potential as special case ν=12 and μ=6. Typically, to describe purely repulsive behavior, i.e., athermal interactions, the Lennard-Jones potential is cut and shifted at its equilibrium, i.e., minimum, position $r^{*}$ [96]. Jover et.al. [97] considered a generalized cut-and-shifted Mie potential:

(A15) $$U_{\lambda_{r},\lambda_{a}}^{\mathrm{hc}}\left(r\right)=\frac{\lambda_{r}}{\lambda_{r}-\lambda_{a}}{\left(\frac{\lambda_{r}}{\lambda_{a}}\right)}^{\frac{\lambda_{a}}{\lambda_{r}-\lambda_{a}}}\in \left\{{\left(\frac{d_{p}}{r}\right)}^{\lambda_{r}}-{\left(\frac{d_{p}}{r}\right)}^{\lambda_{a}}\right\}-\in \;\;\;\;\;\mathrm{for}\;r<r^{*}=d_{p}{\left(\frac{\lambda_{r}}{\lambda_{a}}\right)}^{\frac{1}{\lambda_{r}-\lambda_{a}}},$$

and zero otherwise. Here, ∈ defines the energy scale. The authors in Ref. [97] suggest $\lambda_{r}=50$ and $\lambda_{a}=49$ to be used in molecular dynamics simulation when modeling hard sphere potentials as it proved to yield reasonable results and far more adequately resembles the ideal hard core potential. The form of $U_{50,49}^{\mathrm{hc}}\left(r\right)$ and the cut-and-shifted Lennard-Jones potential ($U_{12,6}^{\mathrm{hc}}\left(r\right)$) are plotted in Figure A1 against the ideal hard core potential drawn as a gray dashed-dotted line. Unfortunately, this form $U_{50,49}^{\mathrm{hc}}\left(r\right)$ has two disadvantages when applying in our approach. Firstly, it contains an uneven power function, $\lambda_{a}=49$, which requires the computation of the square root of $r^{2}=r\;\cdot \;r$. Secondly, $U_{50,49}^{\mathrm{hc}}\left(r\right)$ raises very steeply in the onset of repulsive interaction ($r≲r^{*}$) requiring very small iteration steps to prevent ’breakdowns’ or ’bursts’ of the algorithm as two particles are approaching each other. Thus, for our purposes, the $U_{50,49}^{\mathrm{hc}}\left(r\right)$ would drastically slow down the computation performance and we suggest an alternative form $U_{180,2}^{\mathrm{hc}}\left(r\right)$ which copes with our requirements and at the same time it likewise resembles the ideal hard core repulsion as can be seen from Figure A1.

The corresponding force $f_{i}^{\mathrm{hc}}$ for each inclusion *i* is then constructed straightforwardly:

(A16) $$f_{i}^{\mathrm{hc}}=-\sum_{j\neq i}^{N}\nabla_{i}U_{180,2}^{\mathrm{hc}}\left(r_{ij}\right).$$

The contribution $f_{i}^{\mathrm{th}}$ refers to thermal or Brownian motion of the particles and can be disregarded due to the comparatively large size of the inclusions $d_{p}∼5\;m$ and the condition that they stick to an elastic matrix (i.e., no free motion). The energy $\Delta U^{\mathrm{el}}$ to deflect a particle from its equilibrium position by some Δr can be estimated in leading order as $\Delta U^{\mathrm{el}}∼k\Delta r^{2}$. Equating this elastic energy with the typical order of thermal energy (∼$k_{B}T$), we conclude that, due to thermal fluctuations, the corresponding deflections are of order $\Delta r∼\left(10^{-4}-10^{-5}\right)d_{p}$ considering typical values for the elastic modulus E∼ (1–500) kPa. Thus, we neglect $f_{i}^{\mathrm{th}}$ in Equation (A12).

The last term on the left side of Equation (A12), $f_{i}^{\mathrm{fr}}$, refers to viscous friction when a particle is dragged through the embedding medium upon local repositioning. Primarily, this term defines the dynamical evolution of the rearrangement processes and accounts for the dissipation of kinetic energies. Its existence assures the particles to come to rest at some ’new’ equilibrium positions after a typical time scale associated with the viscous properties of the system. Analogue to the linear elasticity model, we also consider the viscous properties of the matrix as linear, i.e., Newtonian fluid. Accordingly, in the leading order, we describe the viscous friction of a particle moving through the matrix via Stokes’ law:

(A17) $$f_{i}^{\mathrm{fr}}=-b{\dot{r}}_{i}=-3\pi \eta d_{p}{\dot{r}}_{i}.$$

Here, η denotes the viscosity parameter and we introduced the friction constant $b:=3\pi \eta d_{p}$. Typically, for polymer melts, we have η∼10 kPas. Since the chains are additionally crosslinked, we can safely assume that such η represents a lower limit for our system. We note that, already for η∼10 kPas, the dynamics are highly overdamped. For example, the time scale Δt until a particle reaches the limiting overdamped velocity ${\dot{r}}_{\infty }$ can be estimated as $\Delta t∼\frac{m}{b}∼\frac{\rho_{\mathrm{Fe}}\pi d_{p}^{2}}{18\pi \eta }∼10^{-12}s$, where we exemplary utilize the mass density of iron $\rho_{\mathrm{Fe}}$. Considering the magnetic dipole-dipole forces as typical ’drag’ forces acting on the particles, we can evaluate the typical distance Δr a particle moves within such time scale to reach ${\dot{r}}_{\infty }$ as $\Delta r≲10^{-7}d_{p}$. Alternatively, we can take recourse from well-known relations for an overdamped harmonic oscillator upon considering the particles embedded in the linear elastic medium as pinned to a Hookean spring with constant *k*. Then, the condition for overdamped oscillations reads $b^{2}>4km$ which requires $\eta^{2}>\frac{2}{27}Ed_{p}^{2}\rho_{\mathrm{Fe}}$. Apparently, for E∼100 kPa and η∼10 kPas, this holds by several orders of magnitude, $10^{8}\gg 10^{3}$. Therefore, we may describe the motion of embedded particles as strongly overdamped and safely neglect the inertia term, i.e., the right-hand side, in Equation (A12).

Finally, the model discussed here with its corresponding parameters, i.e., micron-sized inclusions in linear elastic medium, yields an equation of motion for each particle as dependent on the actual distribution of the other particles. Since in good approximation we can consider the motion as strongly overdamped, it represents a first order differential equation:

(A18) $$b{\dot{r}}_{i}=F_{i}:=f_{i}^{d}+f_{i}^{\mathrm{el}}+f_{i}^{\mathrm{hc}}.$$

We introduce the dimensionless time scale $T:=\frac{k}{b}t=\frac{E}{3\eta }t$ and the dimensionless length scale $R=\frac{1}{d_{p}}r$. Thus, parameter η, which so far has only been specified by its rough order of magnitude η≳10kPas, is adsorbed in the timescale *T* and is no longer a relevant parameter for the simulation. It does not affect the rearrangement process when referring to dimensionless times *T*. With respect to the real time scale, i.e., *t*, the value of η rather universally tunes the speed of the rearrangement process. The remaining relevant forces, i.e., magnetic, elastic and hard-core repulsion, are summed in $F_{i}$. To approximate the time evolution of the system, we apply a one-step discretization with fixed step-width $\Delta T=T^{n}-T^{n-1}\;,\forall n$ and find the following iteration scheme:

(A19) $$R_{i}^{n+1}=R_{i}^{n}+\Delta T\frac{F_{i}\left(\left\{R^{n}\right\}\right)}{kd_{p}}.$$

In the Supplementary Materials, we attached three example videos to visualize rearrangement processes as computed by the simulation approach suggested here.
