# Supplementary material for: Magneto-Mechanical Coupling in Magneto-Active Elastomers
Source: Materials (Basel). 2021 Jan 17;14(2):434. doi: 10.3390/ma14020434 (PMC7830580; doi:10.3390/ma14020434)
Supplement: Supplementary file 1 [file materials-14-00434-s001.zip › SupplInf.pdf]

# Magnetomechanical Coupling in Structured Magnetic Elastomer: Supplementary Information

**Philipp Metsch, Dirk Romeis, Karl A. Kalina, Alexander Raßloff, Marina Saphiannikova and Markus Kästner**

The videos provided in the Supplementary Information have been visualized using OVITO (A. Stukowski, Modelling Simul. Mater. Sci. Eng. 18, 015012 (2010)). The simulations where performed according to the model as described in Appendix B of the main article. At time step 'zero' an external field of $B^{\infty} = 1\text{T}$ is applied instantaneously along the $x$-axis, i.e. from left to right, and the videos display the subsequent rearrangement process. The parameter sets are the following:

- Video 1: 7 particles arranged in helical chain with rotational angle $\Delta\vartheta = 60^{\circ}$ at radius-to-distance ratio $\alpha = r_c/b = 0.25$ in an elastic matrix with modulus $E = 100\text{kPa}$. Time step is $\Delta T = 4 \cdot 10^{-5}$.
- Video 2: Same as video 1, but in an elastic matrix with modulus $E = 40\text{kPa}$. The playback speed is doubled compared to video 1. Thus, the rearrangement process takes considerably longer compared to $E = 100\text{kPa}$ in video 1.
- Video 3: 75 particles distributed randomly inside an imaginary cubic cell such that the volume fraction of particles is initially $\phi = 15\%$ inside the cell. The elastic modulus of the matrix is set to $E = 15\text{kPa}$ to have large effects (for $E = 100 - 200\text{kPa}$ almost no motion is visible). The playback speed is tuned by factor 20 compared to video 1. We note that, according to simulations via the here developed model, that translated to real time scale, assuming a viscosity of $\eta \sim 10\text{kPas}$, rearrangement processes in (very) soft matrices easily reach the range of seconds.
